# Fear of hypoglycaemia among patients with type 2 diabetes mellitus: a cross-sectional study

**DOI:** 10.1038/s41598-021-86954-0

**Published:** 2021-04-12

**Authors:** Yanhao Wang, Zihuan Zeng, Jie Ding, Ruizhu Yuan, Ruiding Wang, You Zhang, Liyao Bai, Huan Yu, Jiao Tang

**Affiliations:** 1 Key Laboratory of Shaanxi Province for Craniofacial Precision Medicine Research, College of Stomatology, Xi’an Jiaotong University, 98 XiWu Road, Xi’an, 710004 Shaanxi China; 2 School of Nursing, Chongqing Medical University, 1#, Medical College Road, Chongqing, 400016 China; 3 Department of Orthodontics, Xi’an Jiaotong University School of Stomatology, 98# Xiwu Road, Xi’an, 710000 Shaanxi China; 4 College of Stomatology, Chongqing Medical University, Chongqing, China; 5 School of Foreign Languages, Chongqing Medical University, Chongqing, China

**Keywords:** Endocrine system and metabolic diseases, Preventive medicine

## Abstract

To investigate the fear of hypoglycaemia in patients with type 2 diabetes mellitus (T2DM), to identify factors related to this fear, and thus to provide evidence for clinical assessment. A total of 385 patients with T2DM who were admitted to the departments of endocrinology in five tertiary grade-A hospitals in Chongqing, China were included in this study. A questionnaire for general information and a Chinese version of Hypoglycemia Fear Survey (HFS) were used to collect the data. The average total score on the HFS was 71.67 ± 17.06 (HFS-W was 38.15 ± 10.57; HFS-B was 33.52 ± 9.54).The three items with the highest average score for HFS-W were not recognising low blood glucose (BG), not having food available, experiencing a hypoglycaemic episode alone, and for HFS-B were eating large amount of snacks, measuring BG six or more times per day, and keeping BG > 150 mmol/L. Regressions showed that number of hospitalisations for T2DM, receiving health education on diabetes, age and hypoglycaemia history because of T2DM were associated with fear of hypoglycaemia (all *p* < 0.05). Fear of hypoglycaemia in hospitalised patients with T2DM was strongly associated with diabetes health education, hospitalisation for diabetes, age, and hypoglycaemia history. Medical professionals should attach importance to the specific psychological interventions, health education on diabetes and the early prevention of hypoglycaemia or diabetic complications for patients with T2DM to reduce the fear of hypoglycaemia and improve their health status.

## Introduction

Since the 1980s, nearly all the countries in the world have experienced a rapid increase in the number of patients with type 2 diabetes mellitus (T2DM), particularly several developing countries such as China^1–4^. According to the latest statistics, 9.3% of adults aged between 20 and 79 all around the world are living with diabetes^5^. At present, China has the largest number of patients with diabetes throughout the world (about 116.4 million) with its total diabetes-related health expenditure in 2019 reaching the equivalent of $109 billion. The rapidly growing number of patients with diabetes in China is estimated to exceed 140.5 million in 2035, creating a greater burden^5^. The primary purpose of diabetes treatment and care is to achieve optimal blood glucose control and prevent multiple complications of diabetes^3,6,7^. Intensive treatment of diabetes can significantly reduce the progression of microvascular and macrovascular complications^8^.

However, the occurrence of hypoglycaemia induced by intensive glycemic control has become a major obstacle in diabetes treatment, which not only has negative effects on the body^9^ but also causes psychological burdens^10,11^. Usually, hypoglycaemia is unpredictable and can occur during the day or at night. Severe hypoglycaemia has been reported as a cause of death in patients with diabetes^12–16^. Therefore, the discomfort brought by hypoglycaemia and the potential threat to life induce the patient to fear hypoglycaemia (FoH)^17^. Although advances have been made in techniques (e.g., insulin analogues) and evidence-based diabetes management, FoH remains a problem in diabetes management^18^. FoH may change the way patients behave, making them more likely to engage in overcompensating behaviours, such as increasing food intake, reducing the use of insulin, monitoring blood glucose frequently, etc. These behaviours are intended to keep blood glucose (BG) levels above the target range to minimise the frequency and severity of hypoglycaemia^17,19^. However, these behaviours may lead to poor treatment compliance and glycemic control, which may cause negative effects (e.g., reducing quality of life^20^, impairing glycemic control^21^, and subsequent development of microvascular complications^22^) on diabetes treatment. Therefore, reducing FoH level of the patients is the key to avoiding overcompensating behaviours and to facilitating adherence to diabetes treatment.

Previous studies have shown that FoH is related to anxiety and depression in patients^23^. Moreover, it was confirmed that FoH could have a profound impact on glucose control and diabetes treatment in patients^4^. However, to our knowledge, in mainland China, few studies have focused on FoH in patients with T2DM. Therefore, it is necessary to study FoH among patients with diabetes. The purpose of this study was to investigate the current status of FoH and identify its related factors among patients with T2DM. The findings of this study may provide resources for the development of diabetes management.

## Methods

### Participant selection

This was a multicentre cross-sectional survey. The study was conducted in Chongqing, a municipality directly administered by the central government of China. Multicentre convenience sampling was used to select patients from the department of endocrinology in five tertiary grade-A hospitals in Chongqing, China.

Inclusion criteria of this study were: (1) meeting WHO diagnostic criteria for T2DM; (2) aged ≥ 18; (3) diabetes duration ≥ 1 year; (4) good cognitive and literacy skills; (5) volunteering for this study. Exclusion criteria were: (1) Type 2 diabetes with pregnancy, malignant tumour, or other serious complications; (2) History or family history of mental illness; (3) Other serious conditions (such as blindness, paralysis, amputation, consciousness disorder, etc.), which lead to difficulty in conversation and writing. The sample size was estimated according to the equation n ≥ 10 × m, where m is the number of independent variables to be included in the planned linear regression^24^. In this study, there were 15 independent variables, so the calculated sample size is at least 150.

### Data collection

The data were collected between January and December 2019 by five investigators. Before the survey began, investigators had been given uniform guidance and training. During the survey, the investigators first explained the study purpose and procedure to the patients. Patients who agreed to participate were asked to sign a consent form. Then the investigators used unified guidance statements to guide the patients to fill the scale by themselves. To ensure the authenticity of the data, the questionnaire was administered and collected on the spot. In collecting process, every questionnaire was checked item by item. If missing information were found, the investigator would remind the participants to check the questionnaire and ask them to complete it. The questionnaire was collected after being checked again.

#### Survey instrument

The questionnaire used in this study was composed of a general information questionnaire and Hypoglycaemia Fear Survey (HFS). The general information questionnaire consists of two sections: demographic part (e.g., age, sex, income status, etc.) and clinical (disease-related) part (e.g., disease course, family history, treatment methods, etc.).

The original HFS questionnaire was developed in 1987 by Prof. Cox of the University of California, with the purpose of assessing the fear of hypoglycaemia and the related behaviours of patients with diabetes. Afterward, the author revised the scale and developed the commonly used HFS-II^22,23,25^. HFS-II has 33 items and two subscales: worry subscale (HFS-W) and behaviour subscale (HFS-B), covering fear of the consequences of hypoglycaemia for the former part and the measures taken to prevent hypoglycaemic episodes in the latter part. The items in HFS-W part measure worry about hypoglycaemia and its negative effects (18 items), and the items in HFS-B part measure the behaviours to avoid hypoglycaemia and its negative consequences (15 items)^25^. The participants were asked to indicate their fear of hypoglycaemia in each of the 33 items on 5-point Likert-type HFS from 0 (never) to 4 (always). The higher the score, the greater the fear or the more significant change in behaviours are supposed to be^26,27^. The original HFS-II has been translated into Chinese with reported adequate validity and reliability^26,27^. In our study, the Cronbach's coefficient of HFS-II was 0.888 and the Cronbach's coefficient of HFS-B and HFS-W were 0.832 and 0.868, respectively.

### Ethical statement

The procedures of this study were in accordance with Declaration of Helsinki. The protocol of this study was approved by the Ethics Committee of Chongqing Medical University. Before the investigation began, the participants signed the informed consent.

### Statistical analysis

SPSS software (version 25.0) was used for data analysis in this study. Frequency and proportion were used for describing categorical variables, and mean (SD) or median (IQR) were obtained to describe continuous variables as appropriate (mean (SD) was used to express the measurement data fitting the normal distribution, otherwise median and quartile were used). For univariate analysis, the independent-samples t-test or one-way analysis of variance was used to analyse the data fitting the normal distribution and homogeneous variances, and the Kruskal Wallis H (K) test was used to analyse the data fitting the skewed distributions or unequal variances as appropriate. Variables with statistical significance in univariate analysis were included in multivariate linear regression for multivariate analysis. Bilateral *p* < 0.05 indicates statistical significance.

## Results

### Sample characteristics

During the recruitment period a total of 426 eligible patients were screened, among whom 385 accepted the invitation to participate in the study, and the response rate was 90.4%. The average age of the participants was 57.65 ± 15.15; 188 (48.8%) of them were above 60 years old, 196 (50.9%) participants were female, 297 (77.1%) participants were hospitalised at least twice because of diabetes, and 239 (62.1%) had received health education about diabetes before (Table 1).

**Table 1:** Sociodemographic and clinical characteristics of type 2 diabetic patients (*n* = 385).

Characteristic		N	%
**Gender**			
Male		189	49.1
Female		196	50.9
**Age (years)**			
Mean (SD) = 57.65 (15.15)			
< 60		197	51.2
≥ 60		188	48.8
**Marital status**			
Married		334	86.8
Unmarried		51	13.2
**Employment status**			
Employed		137	35.6
Unemployed		248	64.4
**Educational attainment**			
Primary or middle school		105	27.3
High school		184	47.8
College or above		96	24.9
**Times of hospitalization because of T2DM**			
First time		88	22.9
Second time or more		297	77.1
**Receiving health education on diabetes**			
Yes		239	62.1
No		146	37.9
**Hypertension**			
Yes		181	47.0
No		204	53.0
**Hyperlipidaemia**			
Yes		132	34.3
No		253	65.7
**Hypoglycaemia history**			
Yes		222	57.7
No		163	42.3
**Mastering the knowledge of hypoglycaemia prevention and management**			
Yes		108	28.1
No		277	71.9
**Duration since diagnosis (years)**			
Mean (SD) = 9.93 (7.54)			
< 5		122	31.7
5–9.9		103	26.7
≥ 10		160	41.6
**Diabetes treatment type**			
Oral medication		118	30.6
Insulin		66	17.2
Oral medication + insulin		201	52.2
**Body mass index (kg/m**^**2**^**)**			
Mean (SD) = 24.06 (3.22)			
< 18.5		12	3.1
18.5–23.9		176	45.7
24–27.9		157	40.8
≥ 28		40	10.4
**Haemoglobin A1c (%) (n = 363)**			
Mean (SD) = 10.32 (2.02)			
< 7		14	3.9
≥ 7		349	96.1

SD, standard deviation; T2DM, type 2 diabetes mellitus.

### Fear of hypoglycaemia

The average total score of HFS was 71.67 ± 17.06 (HFS-W was 38.15 ± 10.57 and HFS-B was 33.52 ± 9.54), the average score for a single item of HFS-W and HFS-B was 2.12 ± 0.59 and 2.23 ± 0.64, respectively. The three items with the highest average score for HFS-W were not recognising low BG, not having food available, and experiencing a hypoglycaemic episode alone (Table 2), for HFS-B eating large amount of snacks, measuring BG six or more times per day and keeping BG > 150 mmol/L (Table 3).

**Table 2:** Fear of hypoglycaemia (Worry subscale) in type 2 diabetic patients (*n* = 385).

Fear of hypoglycemia	Mean (SD)
HFS-W	2.12 (0.59)
HFS-W1 Not recognizing low BG	2.41 (1.13)
HFS-W2 Not having food available	2.39 (1.05)
HFS-W5 Experiencing hypoglycaemic episode alone	2.37 (1.11)
HFS-W13 Feeling lightheaded or dizzy	2.33 (1.09)
HFS-W3 Passing out in public	2.31 (1.09)
HFS-W15 Permanent injury to health	2.19 (1.12)
HFS-W10 Making mistakes or having accidents	2.14 (1.07)
HFS-W18 Becoming upset and difficult	2.12 (1.08)
HFS-W8 No one to help during hypoglycaemia	2.11 (1.09)
HFS-W12 Difficulty thinking clearly	2.09 (1.03)
HFS-W16 Low BG interfering with important things	2.08 (1.03)
HFS-W6 Appearing drunk or stupid	2.03 (1.04)
HFS-W4 Embarrassing myself in social situation	2.03 (1.03)
HFS-W14 Injuring myself or others	2.00 (1.02)
HFS-W7 Losing control	1.97 (1.02)
HFS-W17 Becoming hypoglycaemic while sleeping	1.94 (1.06)
HFS-W11 Getting a bad evaluation	1.94 (1.00)
HFS-W9 Having hypoglycaemia while driving	1.71 (0.99)

SD, standard deviation; HFS-W, worry subscale of hypoglycaemia fear survey; BG, blood glucose.

**Table 3:** Fear of hypoglycaemia (Behaviour subscale) in type 2 diabetic patients (*n* = 385).

Fear of hypoglycaemia	Mean (SD)
**HFS-B**	**2.23 (0.64)**
HFS-B1 Ate large snack	2.71 (1.23)
HFS-B4 Measured BG six or more times per day	2.53 (1.15)
HFS-B2 Kept BG > 150 mmol/L	2.40 (1.13)
HFS-B10 Limited physical activity	2.23 (1.19)
HFS-B8 Avoided visiting friends	2.21 (1.15)
HFS-B5 Take someone with me when out	2.21 (1.08)
HFS-B7 Limited driving	2.19 (1.31)
HFS-B14 Kept BG high during important tasks	2.16 (1.19)
HFS-B12 Avoided sex	2.15 (1.14)
HFS-B3 Reduced insulin when BG low	2.13 (1.15)
HFS-B6 Limited out-of-town travel	2.12 (1.19)
HFS-B9 Stayed home more than liked	2.08 (1.10)
HFS-B13 Kept BG high in social situation	2.05 (1.14)
HFS-B15 Had someone else check on me	2.05 (1.11)
HFS-B11 Made sure others were around	2.01 (1.07)

SD, standard deviation; HFS-B, behaviour subscale of hypoglycaemia fear survey; BG, blood glucose.

### Factors related to fear of hypoglycaemia

Univariate analysis identified factors that were significantly associated with the average total score of HFS of the patients. In univariate analysis, the average total score of HFS was higher in patients who were employed (t = 2.603, *p* = 0.010), age more than 60 years (t = 3.292, *p* = 0.001), hospitalised because of diabetes for the first time (t = 4.806, *p* < 0.001), without hyperlipidaemia (t = 2.399, *p* = 0.017), whose educational attainment was college or above (F = 6.368, *p* = 0.002), who had a history of hypoglycaemia (t = − 3.428, *p* = 0.001), mastered the knowledge of hypoglycaemia prevention and management (t = − 2.730, *p* = 0.007), and received health education on diabetes (t = − 5.291, p < 0.001) (Table 4).

**Table 4:** Differences in fear of hypoglycaemia among various sociodemographic and clinical subgroups (*n* = 385).

Variable	*n*	Mean (SD)	*t/F*	*P*
**Age**			3.292	0.001
< 60	197	74.44 (16.21)		
≥ 60	188	68.77 (17.49)		
**Employment status**			2.603	0.010
Employed	137	74.69 (14.36)		
Unemployed	248	70.00 (18.20)		
**Educational attainment**			6.368	0.002
Primary school	105	69.59 (18.28)		
High school	184	70.09 (16.72)		
College or above	96	76.97 (15.32)		
**Number of hospital admissions because of T2DM at enrollment**			4.806	0.000
First time	88	79.14 (11.38)		
Second time or more	297	69.46 (17.84)		
**Receiving health education on diabetes**			− 5.291	0.000
Yes	239	75.15 (15.42)		
No	146	65.98 (18.12)		
**Hyperlipidaemia**			2.399	0.017
Yes	132	68.83 (16.54)		
No	253	73.15 (17.18)		
**Hypoglycaemia history**			− 3.428	0.001
Yes	222	74.22 (16.31)		
No	163	68.20 (17.50)		
**Mastering the knowledge of hypoglycaemia prevention and management**			− 2.730	0.007
Yes	108	75.34 (16.18)		
No	277	70.24 (17.21)		
**Duration since diagnosis**			3.302	0.038
< 5	122	73.45 (16.28)		
5–9.9	103	73.65(16.09)		
≥ 10	160	69.04 (17.99)		

SD, standard deviation; T2DM, type 2 diabetes mellitus.

Then the variables with statistical significance in univariate analysis were included in multivariate linear regression for multivariate analysis. Table 5 presents the results of the found in patients who were hospitalized for the first time because of T2DM (B = − 8.434, *p* < 0.001), were younger (B = − 0.170, *p* = 0.002), had a history of hypoglycaemia (B = 4.163, *p* = 0.018), and received health education on diabetes (B = 6.263, *p* = 0.001). The independent variables accounted for 18.3% of hypoglycaemia fear in patients with T2DM (Table 5).

**Table 5:** Factors related to fear of hypoglycaemia among type 2 diabetic patients (*n* = 385).

Variable	*B*	*SE-B*	*β*	*t*	*P*
Constant	79.694	5.575	–	14.295	0.000
Number of hospital admissions because of T2DM at enrollment^**†**^	− 8.434	1.968	− 0.208	− 4.286	0.000
Receiving health education on diabetes^**†**^	6.263	1.817	0.178	3.447	0.001
Age^**†**^	− 0.170	0.056	− 0.151	− 3.062	0.002
Hypoglycaemia history^**†**^	4.163	1.754	0.121	2.374	0.018

B, unstandardized coefficient beta; SE-B, standard error of B; β, standardized coefficient beta; T2DM, type 2 diabetes mellitus.

^**†**^Number of hospital admissions because of T2D at enrollment: 1 = First time, 2 = Second time or more; Receiving health education on diabetes: 1 = No, 2 = Yes; Hypoglycaemia history: 1 = No, 2 = Yes.

## Discussion

Evidence showed that FoH had a profound impact on glucose control and diabetes treatment in patients^4^. Reducing the FoH level of the patients with T2DM is the key to avoiding overcompensating and facilitating diabetes control. In this study, the average total score on the HFS was 71.67 ± 17.06 (HFS-W was 38.15 ± 10.57 and HFS-B was 33.52 ± 9.54), which was higher than in some previous studies^26–28^. The potential risk of hypoglycaemia may greatly affect patients’ work and daily life, making them feel distressed and anxious^29^. Moreover, the participants in this study were all hospitalised patients with T2DM whose conditions may be worse than those of non-hospitalised patients with T2DM. Therefore, the FoH of participants in this study may be higher than that of the participants (non-hospitalised patients with T2DM were included) in the study of Gonder-Frederick et al.^28^.

The three items with the highest average score for HFS-W were not recognising low BG, not having food available, experiencing hypoglycaemic episode alone, and the three items with the highest average score for HFS-B were eating large amount of snacks, measuring BG six or more times per day, and keeping BG > 150 mmol/L. According to the above results, it is more likely that lack of knowledge or specific measures to prevent and relieve hypoglycaemia can lead to fear in patients or make them behave in a way that is not conducive to the treatment of diabetes. Medical professionals can effectively reduce the FoH of patients through strengthening their knowledge about the identification and prevention methods of hypoglycaemia by multiple means of health education (e.g., peer coaches^30^, empowerment-based health education^31^, diabetes case management intervention^32^, etc.), preparing adequate glucose solutions, encouraging their family members to do more to help patients with T2DM to control BG, and giving patients more comprehensive dietary, medication, and self-management guidance.

The patients who were hospitalised for the first time for diabetes had a higher FoH level than these who were hospitalised because of diabetes for the second time or more. One explanation for this could be that as the experience in disease management of patients continues to accumulate with the number of hospitalisations, confidence for controlling BG also increases and FoH was then diminished.This finding is similar to the study of Belendez et al.^33^ that found that the FoH level might decrease with the progression of diabetes. However, some studies showed no correlation or positive correlation between FoH level and the course of disease^26,34^. The positive correlation between FoH level and the course of disease may be the result of diabetes, multi-organ complications, or relapse of diabetes that can increase the economic burden for patients and thus aggravate their fear^26,34^. Different findings on the same problem suggest that more studies are needed to further explore the relationship between the progression of diabetes and FoH. In addition, the FoH score of patients hospitalised because of diabetes for the second time or more was also higher than the score of participants in a similar study^28^, which suggests that hospitalised patients should be assessed for fear of hypoglycaemia and treated with timely psychological interventions.

Our study also found that hypoglycaemia history and patient age were factors related to FoH. Patients with a history of hypoglycaemia had higher total FoH scores in the current study. Several studies have shown a close link between hypoglycaemia history and FoH^17,35,36^ and FoH level has had a significant tendency to increase with the severity of hypoglycaemia^36^. It may be that the painful physical and psychological effects of previous episodes of hypoglycaemia may lead to anxiety and fear of hypoglycaemia. Some patients may fear not only the painful consequences of hypoglycaemia but also the symptoms themselves^37^. Moreover, patients with T2DM under the age of 60 had a higher FoH level in our study. The reason might be that the occurrence of hypoglycaemia would seriously affect the work, study, or other sorts of social activities of the sample in this group^26–28^.

However, it is interesting to note that on the basis of the results, receiving health education on diabetes may be a predictor of FoH. Perhaps this is because previous diabetes health education that the participants (Chinese patients with diabetes) received focused on guiding the patients to avoid reducing the BG level by overusing the treatment in order to reduce the incidence of hypoglycaemia^38–40^. Therefore, because health educators may talk about the harm or risk of hypoglycaemia, the patient have different degrees of psychological fear of hypoglycaemia. Although the result relating to health education differs from other studies, it may provide a new avenue for clinical health education. In the future, the harm of hypoglycaemia should not be overemphasised, and the patients should be helped to build confidence in how they can prevent hypoglycaemia. Health education should focus on adjusting the psychological status of patients with diabetes, reducing their fear of hypoglycaemia, and making them aware of the adverse effects on diabetes treatment and blood glucose control of overcompensation behaviours as a result of the fear of hypoglycaemia.

This research has some possible limitations. The first is that we did not use specific monitoring instruments to assess BG in this study. Another limitation is that the cross-sectional design of this study failed to enable testing for responsiveness to changes. More evidence is needed to determine whether such data have sufficient sensitivity to detect change when change is present. Moreover, sociodemographic and clinical variables in this study account for just 18.3% variation of FoH in our multiple linear regression models. Results indicated that there were still some significant related factors not recognised in the present study, and more variables need to be incorporated into the model in further studies.
